# Efficacy and quality of life of Romiplostim in adults and children with immune thrombocytopenia: A review

**DOI:** 10.1097/MD.0000000000032345

**Published:** 2022-12-16

**Authors:** Xin He, Ningyuan Ran, Ting Wang, Zonghong Shao

**Affiliations:** a Department of Hematology, Tianjin Medical University General Hospital, Heping District, Tianjin, China.

**Keywords:** adults and children, health-related quality of life, immune thrombocytopenia, meta-analysis, Romiplostim

## Abstract

To systematically evaluate the clinical efficacy, drug safety and health-related quality of life (HRQoL) of Romiplostim in adult and child immune thrombocytopenia (ITP) patients.

**Methods:**

PubMed, EMBASE and Cohrane library databases were searched for all randomized controlled trials published until 2022, and the Review Manager 5.3 was used for meta-analysis.

**Results:**

A total of 9 randomized controlled trials were included in this study. The results of meta-analysis showed that the total platelet response rate and long-term platelet response rate in treatment group were significantly higher than those in control group (P<0.05). There was no statistical significance in the side effects, serious side effects, bleeding events and serious bleeding events between 2 groups (P>0.05). Compared with control group, the HRQoL in ITP adults and children, and parents of ITP children had no statistical significance (P>0.05).

**Conclusion:**

Romiplostim has a certain clinical efficacy in ITP adults and children, and relatively small adverse drug reactions. The improvement of Romiplostim on HRQoL in ITP adults and children is not clear.

## 1. Introduction

Immune thrombocytopenia (ITP) is an autoimmune disease characterized by bleeding of skin, mucosa and organs. The morbidity of ITP adults is relatively high and most of them are chronic diseases. However, the course of ITP in children is acute, and some children will turn into chronic ITP after treatment.^[1]^ The specific pathogenesis of ITP is not completely clear, mainly due to the over destruction of platelets and insufficient platelet production caused by the abnormal autoimmunity. Therefore, promoting platelet production and preventing platelet over destruction have become important principles in the treatment of ITP adults and children.^[2]^

At present, the first-line treatment of ITP is glucocorticoid and intravenous immunoglobulin, and the second-line treatment is mainly immunosuppressant. In recent years, Some thrombopoietin receptor agonists such as Eltrombopag and Romiplostim have been put into clinical use.^[3]^ Romiplostim is a human TPO mimic peptide produced by recombinant DNA technology. Clinical studies have found that Romiplostim can increase drug efficacy and reduce the incidence of adverse reactions in ITP adults and children.^[4,5]^ In order to further clarify the efficacy and safety of Romiplostim, and evaluate the quality of life in ITP patients. In this study, we collected a number of randomized controlled trials (RCTs) related to Romiplostim in ITP adults and children, and made a meta-analysis from clinical efficacy, drug safety and health-related quality of life (HRQoL). To provide reference and evidence for the clinical application of Romiplostim in the treatment of ITP adults and children.

## 2. Materials and Methods

### 2.1. Articles retrieval

PubMed, EMBASE and Cohrane library databases were searched for all RCTs of Romiplostim in ITP adults and children published until 2022. The search term is: Romiplostim, immune thrombocytopenia, ITP, children with ITP, efficacy, safety, quality of life, etc. At the same time, the references of the retrieved articles is searched once again.

### 2.2. Inclusion and exclusion criteria of articles

#### 2.2.1. Inclusion criteria.

Published RCTs were included in this study to provide complete data for meta-analysis. Patients with confirmed ITP, regardless of age, nationality, sex and race. The experimental group was treated with Romiplostim the control group was treated with placebo. The main outcome of this study were clinical efficacy (total platelet response rate, long-term platelet response rate). The secondary outcome of this study were drug safety (side effects, serious side effects, use of emergency drugs, bleeding events and serious bleeding events) and HRQoL.

#### 2.2.2. Exclusion criteria.

Articles with repeated publications. Retrospective studies, single-arm clinical trial articles, review articles. Articles without full text or incomplete full text data.

### 2.3. Selection of articles

Two professionals independently completed the articles screening. First, search the articles in database, and then read the title and abstract of the articles 1 by 1, excluding the articles that obviously does not meet the inclusion criteria. Next, carefully search and read the full text of these articles that meet requirements, and the articles that are finally determined through the criteria of articles exclusion can be formally included in this study. When there is disagreement in the process of articles selection, it shall be decided by discussion or by a third professional.

### 2.4. Evaluation of articles

Bias risk assessment tool in Revman 5.3 was used to evaluate these included articles. The evaluation mainly includes 6 items: Whether the random grouping is described; Whether the allocation hiding is sufficient; Whether blind is adopted; Whether there is a description of withdrawal from the test and loss of visit; Whether there are outcome for selective reporting; Whether there are other factors that may affect the quality of the study. According to the specific requirements of Cochrane manual for each project, the final judgment of “low risk bias,” “high risk bias” or “unknown risk bias” are obtained. If more than one of the above items is judged as “not low risk bias,” then the test is generally judged as “high risk bias.” When all items are of “low risk bias,” the test is of “low risk bias” in general.

### 2.5. Data extraction

The data of each article is extracted according to the Cochrane Collaboration. The main extracted data include: The subjects, publication time, first author, total sample size, follow-up time and other basic information included in the study; The age, gender, duration of bleeding before admission, platelet count at admission, and loss of visit during withdrawal were included in the study; The intervention measures included name, dose, treatment course and administration mode of the drugs used in the study; Some observation indexes and end-point indexes of each group, such as clinical efficacy (total platelet response rate, long-term platelet response rate), drug safety (side effects, serious side effects, use of emergency drugs, bleeding events and serious bleeding events) and HRQoL. The data were extracted by 2 professionals independently, in case of disagreement, it shall be decided by discussion or by a third professional.

### 2.6. Statistical analysis

System evaluation software Ravman 5.3 provided by Cochrane Collaboration was used for statistical analysis. Chi-square test was used to analyze heterogeneity of the included articles. When *p*>0.1, *I*^2^<50%, it shows that there was no heterogeneity among the studies, using fixed effect model. When *p*<0.1, *I*^2^>50%, it shows that there was heterogeneity among the studies, and using random effect model. Relative risk (RR) was used as the statistical value of efficacy analysis, and all effects were expressed by 95% confidence interval (CI). The difference was statistically significant when p<0.05. Then according to the characteristics of the study object and the different intervention measures, the sub group analysis was selected again.

## 3. Results

### 3.1. Results of articles

**Figure 1.**
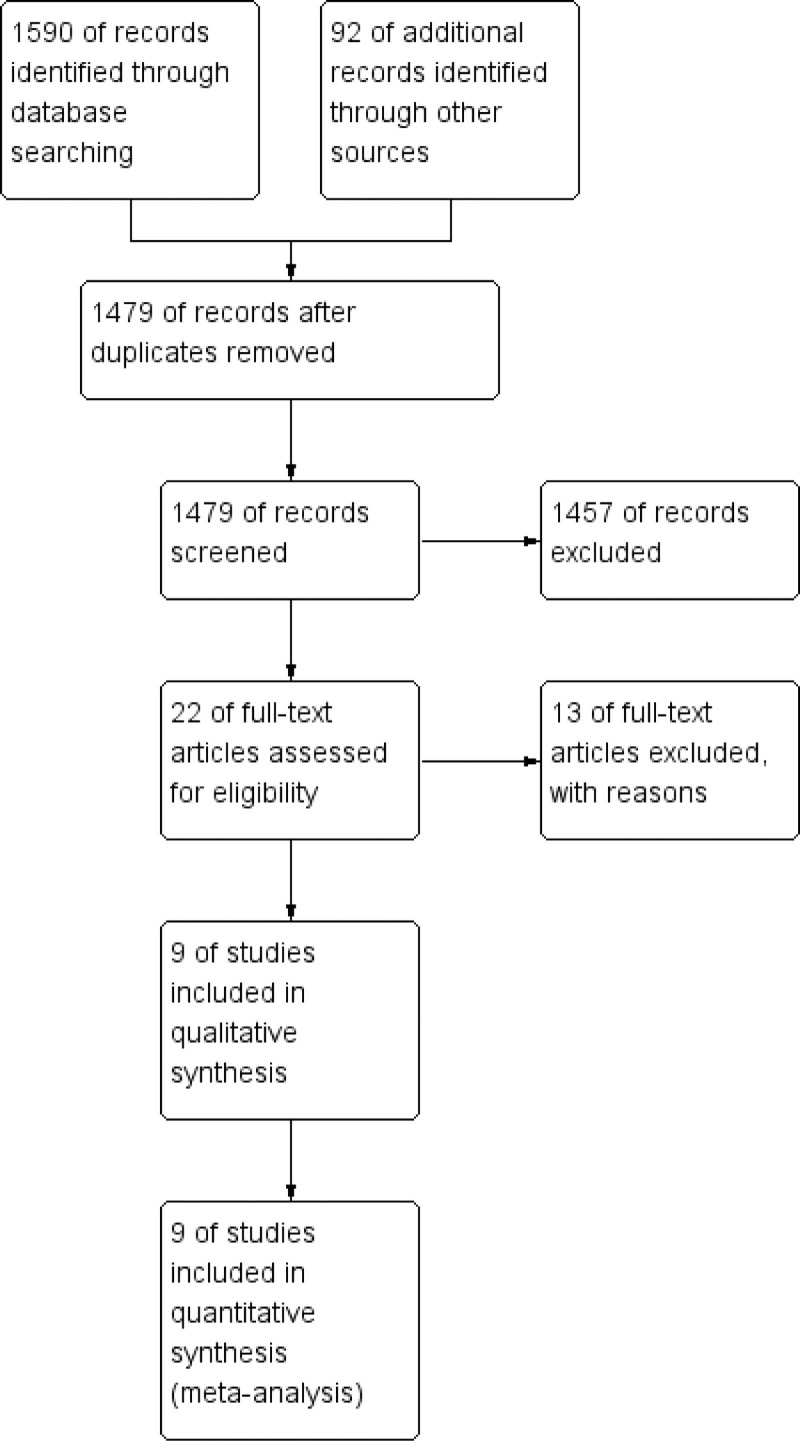
The process of screening articles.

According to the retrieval strategy, 1682 related articles were retrieved from the database. There were 1479 articles left after eliminating the duplicate. After reading the title and abstract of the articles, 22 articles were selected. After reading the full text, 13 articles were excluded. The reasons for exclusion were: 6 non RCTs, 3 repeatedly published articles, 4 post analysis and review articles. Finally, 9 RCTs were included for follow-up analysis.^[6–14]^ The specific screening process is shown in Figure 1.

### 3.2. Basic features of articles

Among the 9 articles finally included, 4 were ITP adults and 5 were ITP children. The sample size of all articles was 275 at most and 18 at least, and the total number of ITP patients was 854. The basic characteristics of the articles are shown in Table 1.

**Table 1:** Basic characteristics of included articles.

**Name of article**	**Content of article**	**Group**	**Number of patients (male/female**)	**Average age (yrs**)	**Treatment**	**Total treatment time**
1. David J Kuter 2008^[6]^	Efficacy and safety in ITP adults	Treatment with Romiplostim after splenectomy	42 (15/27)	51 (27–88)	Weekly hypodermic injection of Romiplostim, the initial dose was 1*μ*g/kg.	24 wks
		Treatment with Placebo after splenectomy	21 (10/11)	56 (26–72)	Weekly hypodermic injection of placebo, the initial dose was 1*μ*g/kg.	
		Treatment with Romiplostim after non splenectomy	41 (14/27)	52 (21–80)	Weekly hypodermic injection of Romiplostim, the initial dose was 1*μ*g/kg.	
		Treatment with Placebo after non splenectomy	21 (5/16)	46 (23–88)	Weekly hypodermic injection of placebo, the initial dose was 1*μ*g/kg.	
2. Yukari Shirasugi 2011^[7]^	Efficacy and safety in ITP adults	Treatment with Romiplostim	22 (8/14)	58.5 (±12.6)	Weekly hypodermic injection of Romiplostim, the initial dose was 3*μ*g/kg.	12 wks
		Treatment with Placebo	12 (2/10)	47.6 (±13.4)	Weekly hypodermic injection of placebo, the initial dose was 3*μ*g/kg.	
3. David J. Kuter 2010^[8]^	Efficacy and safety in ITP adults	Treatment with Romiplostim	157 (72/85)	58 (18–90)	Weekly hypodermic injection of Romiplostim, the initial dose was 3*μ*g/kg.	52 wks
		Treatment with SOC	77 (31/46)	57 (18–86)	Receive the SOC treatment, the initial dose was 3*μ*g/kg.	
		Treatment with Placebo	41 (15/26)	55 (±17)	Weekly hypodermic injection of placebo, the initial dose was 3*μ*g/kg.	
4. Mohsen Saleh Elalfy 2011^[9]^	Efficacy and safety in ITP chlidren	Treatment with Romiplostim	12 (10/2)	9.5 (2.5–16)	Weekly hypodermic injection of Romiplostim, the initial dose was 1*μ*g/kg.	12 wks
		Treatment with Placebo	6 (3/3)	7 (4–15)	Weekly hypodermic injection of placebo, the initial dose was 1*μ*g/kg.	
5. Michael D Tarantino 2016^[10]^	Efficacy and safety in ITP chlidren	Treatment with Romiplostim	42 (18/24)	10 (6–14)	Weekly hypodermic injection of Romiplostim, the initial dose was 1*μ*g/kg.	24 wks
		Treatment with Placebo	20 (9/11)	7.5 (6.5–13.5)	Weekly hypodermic injection of placebo, the initial dose was 1*μ*g/kg.	
6. James B. Bussel 2011^[11]^	Efficacy and safety in ITP chlidren	Treatment with Romiplostim	17 (13/4)	9 (1–17)	Weekly hypodermic injection of Romiplostim, the initial dose was 1*μ*g/kg.	12 wks
		Treatment with Placebo	5 (3/2)	11 (2–14)	Weekly hypodermic injection of placebo, the initial dose was 1*μ*g/kg.	
7. David J. Kuter 2012^[12]^	Quality of life in in ITP adults	Treatment with Romiplostim	157 (72/85)	55 (±19)	Weekly hypodermic injection of Romiplostim, the initial dose was 3*μ*g/kg.	52 wks
		Treatment with SOC	77 (31/46)	55 (±19)	Weekly hypodermic injection of placebo, the initial dose was 3*μ*g/kg.	
8. Susan D. Mathias 2016^[13]^	Quality of life in ITP children	Treatment with Romiplostim	42 (18/24)	9.7 (±4.1)	Weekly hypodermic injection of Romiplostim.	24 wks
		Treatment with Placebo	20 (9/11)	9.4 (±4.7)	Weekly hypodermic injection of placebo.	
9. Robert J.Klaassen 2012^[14]^	Quality of life in ITP children	Treatment with Romiplostim	17 (13/4)	9 (1–17)	Weekly hypodermic injection of Romiplostim, the initial dose was 1*μ*g/kg.	13 wks
		Treatment with Placebo	5 (3/2)	11 (2–14)	Weekly hypodermic injection of placebo, the initial dose was 1*μ*g/kg.	

ITP = immune thrombocytopenia.

### 3.3. Quality evaluation of articles

**Figure 2.**
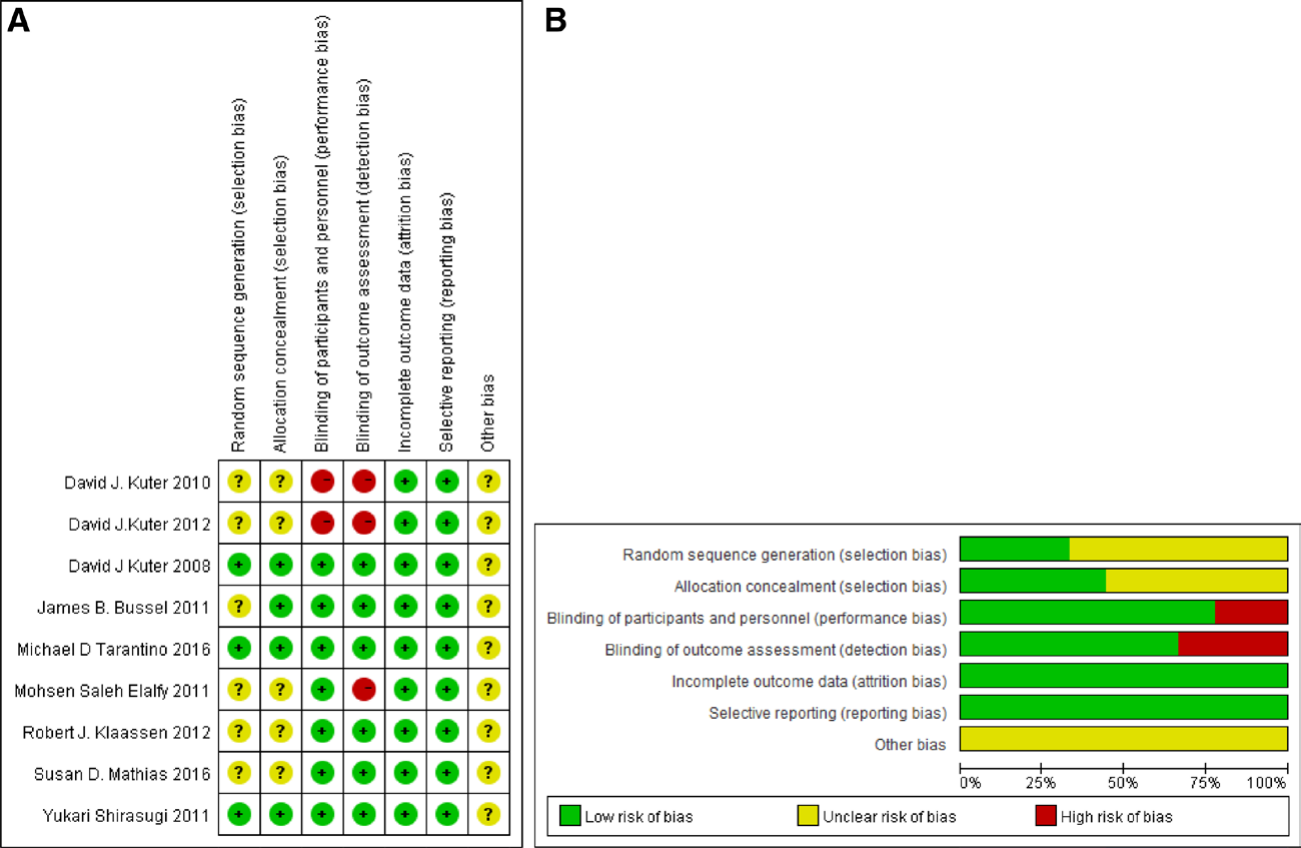
(A): Summary of methodological quality assessment in articles. (B): Proportion of bias risk in articles.

Among the 9 articles included, 6 were double-blind trials, 1 was single-blind trial, and 2 were open trials. 7 articles were treated with placebo, and 2 were treated with SOC. 3 articles described the specific methods of randomized control. All the 9 articles were lost to follow-up, and all of them explained the reasons for their withdrawal or loss. A summary of all the methodological quality assessment is shown in Figure 2A. The proportion of bias risk is shown in Figure 2B.

### 3.4. Meta-analysis of platelet response rate in each group

**Figure 3.**
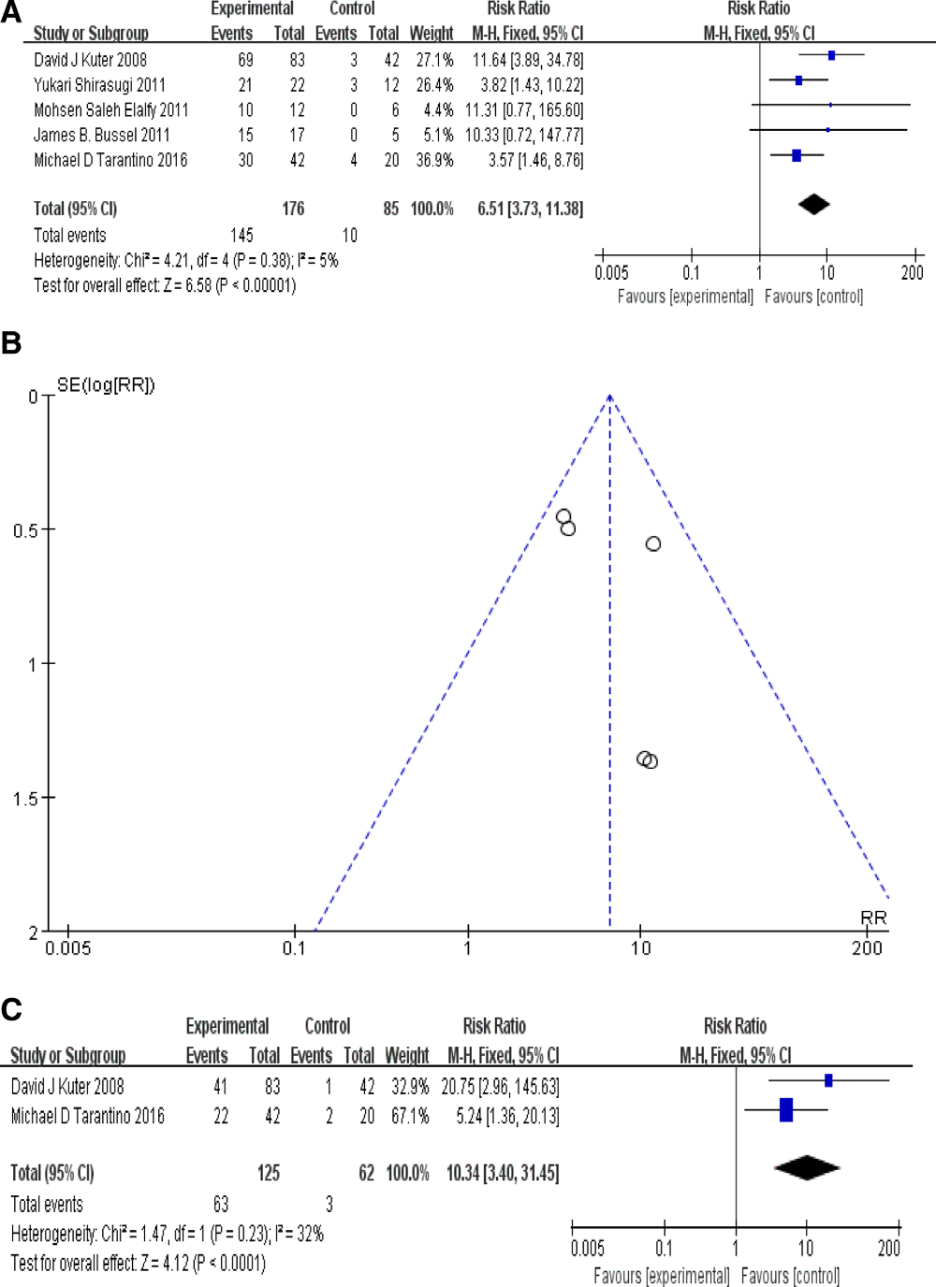
(A): total platelet response rate in each group. (B): Funnel plot of total platelet response rate in each group. (C): long term platelet response rate in each group.

Of the 9 articles included, a total of 5 articles reported the results of total platelet response rate in each group. The heterogeneity among the articles was low (*P* = .38, *I*^2^ = 5%), so we used fixed effect model. Meta-analysis showed that RR = 6.51, 95% CI [3.73, 11.38], *P*<0.01, the difference was statistically significant. As shown in Figure 3A. The funnel plot of total platelet response rate in each group is shown in Figure 3B.

In addition, 2 articles reported the results of long-term platelet response rate in each group. The heterogeneity between the 2 articles was *P* = .23, *I*^2^ = 32%, using fixed effect model. Meta-analysis showed that RR = 10.34, 95% CI [3.40, 31.45], *P*<0.01, the difference was statistically significant. As shown in Figure 3C.

### 3.5. Meta-analysis of drug side effects in each group

**Figure 4.**
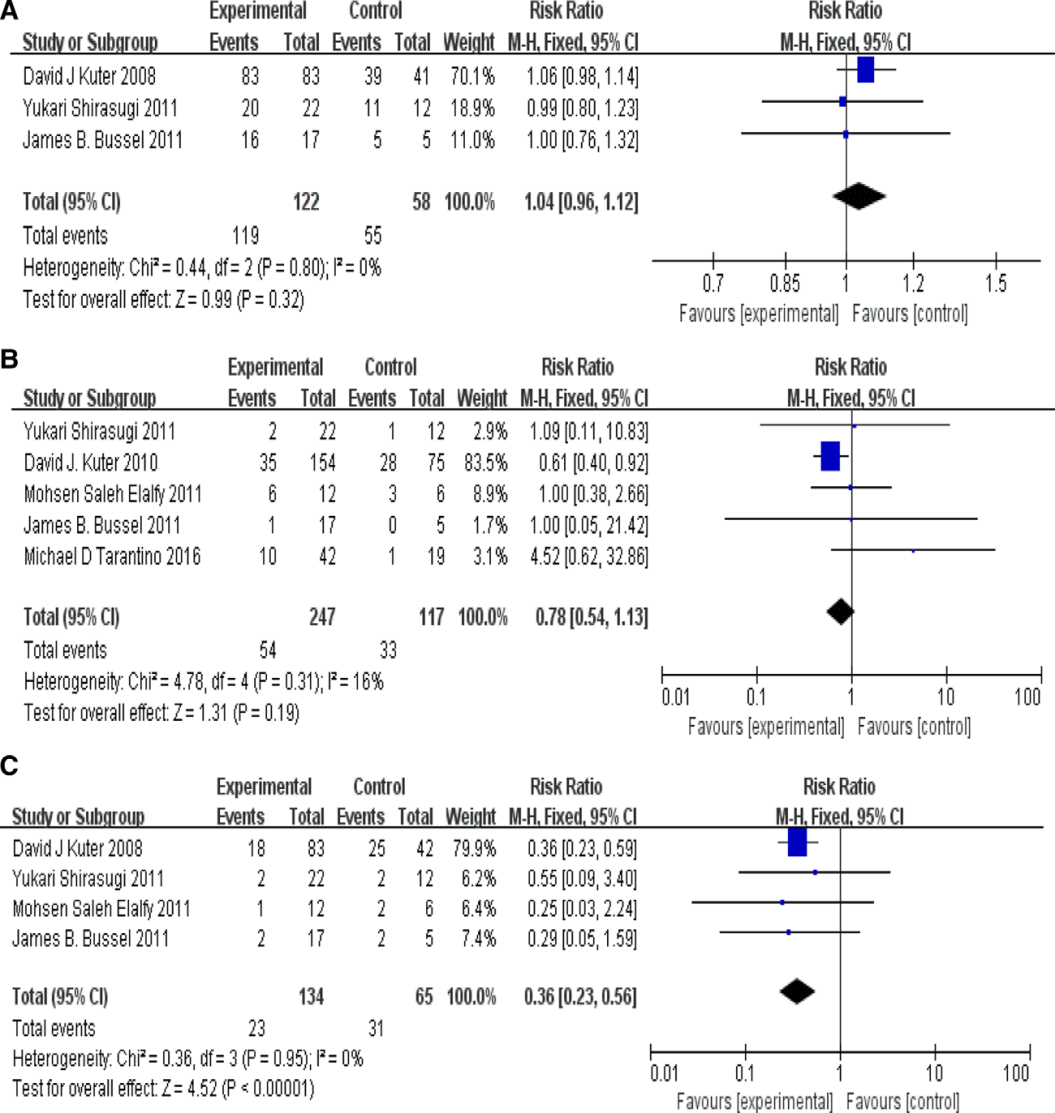
(A): side effects of drugs in each group. (B): serious side effects of drugs in each group. (C): use of emergency drugs in each group.

3 out of 9 articles reported the side effects in each group. There was no heterogeneity among the articles (*P* = .80, *I*^2^ = 0%), and we used fixed effect model. Meta-analysis showed that RR = 1.04, 95% CI [0.96, 1.12], *P*>0.05, so there was no statistical difference in side effects between the groups. As shown in Figure 4A.

There were also 5 articles about the results of serious side effects in each group. The heterogeneity among the articles was small (*P* = .31, *I*^2^ = 16%), and we used fixed effect model. Meta-analysis showed that RR = 0.78, 95% CI [0.54,1.13], *P*>0.05, so there was no significant difference in serious side effects between these groups. As shown in Figure 4B.

In terms of use of emergency drugs, a total of 4 articles reported the results of this in each group. There was no heterogeneity among the articles (*P* = .95, *I*^2^ = 0%), so we used fixed effect model. Meta-analysis showed that RR = 0.36, 95% CI [0.23, 0.56], *P*<0.01, so there was a statistical difference in the use of emergency drugs in each group. As shown in Figure 4C.

### 3.6. Meta-analysis of bleeding events in each group

**Figure 5.**
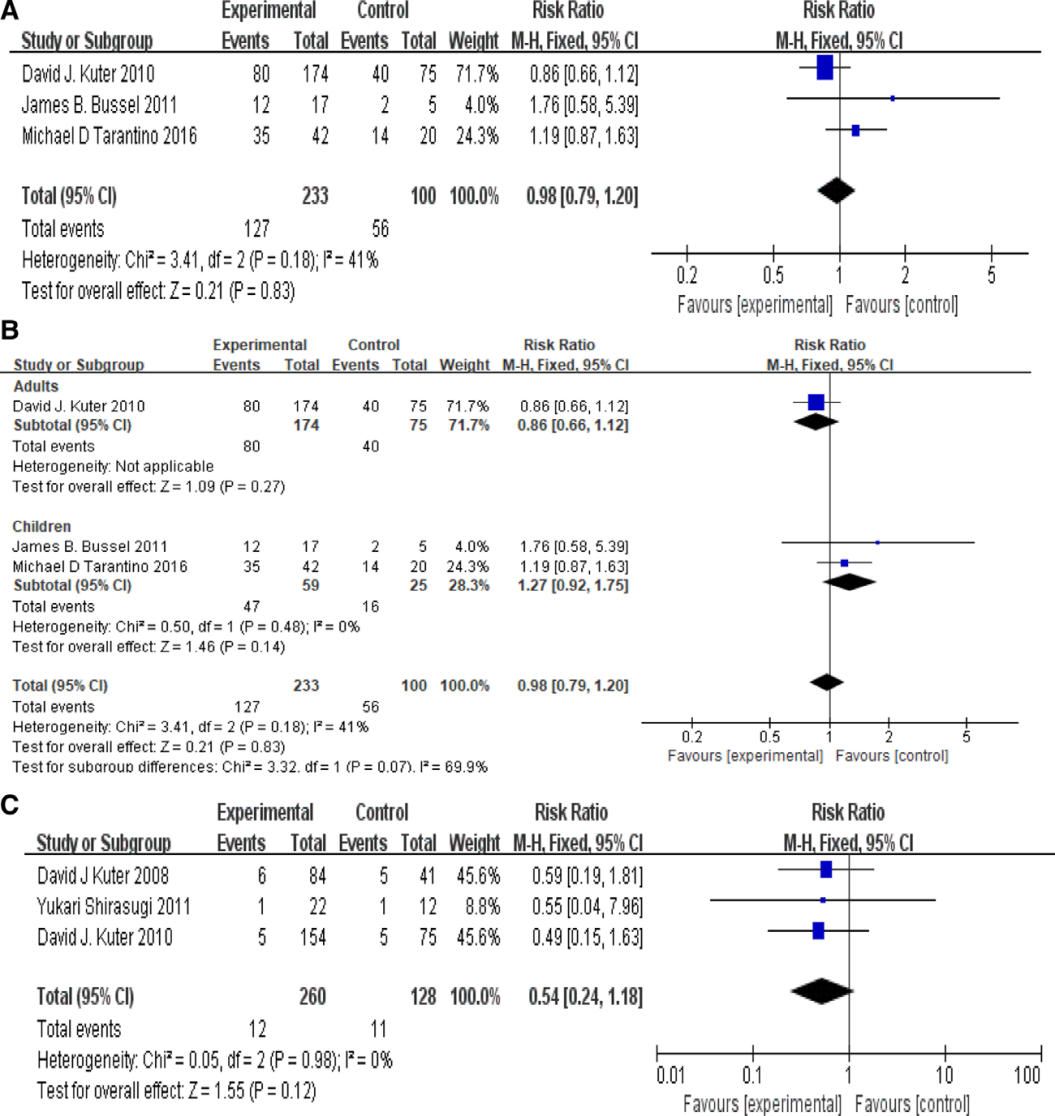
(A): bleeding events occurred in each group. (B): The incidence of bleeding events in ITP adults and children. (C): severe bleeding events occurred in each group. ITP = immune thrombocytopenia.

3 of the 9 articles reported the bleeding events in each group. There was a certain degree of heterogeneity (*P* = .18, *I*^2^ = 41%) among the articles, using fixed effect model. Meta-analysis showed that RR = 0.98, 95% CI [0.79,1.20], *P*>0.05, therefore, there was no statistical difference in the incidence of bleeding events among patients in each group. As shown in Figure 5A. Further subgroup analysis of ITP adults and children was conducted, the results showed that there was no heterogeneity (*P* = .48, *I*^2^ = 0%), and no significant difference in bleeding events among ITP children. As shown in Figure 5B.

In terms of serious bleeding events in each group, 3 articles reported the results. There was no heterogeneity among the articles (*P* = .98, *I*^2^ = 0%). Meta-analysis showed that RR = 0.54, 95% CI [0.24,1.18], *P*>0.05, so there was no statistical difference in the occurrence of serious bleeding events in each group. As shown in Figure 5C.

### 3.7. Meta-analysis of HRQoL in each group

**Figure 6.**
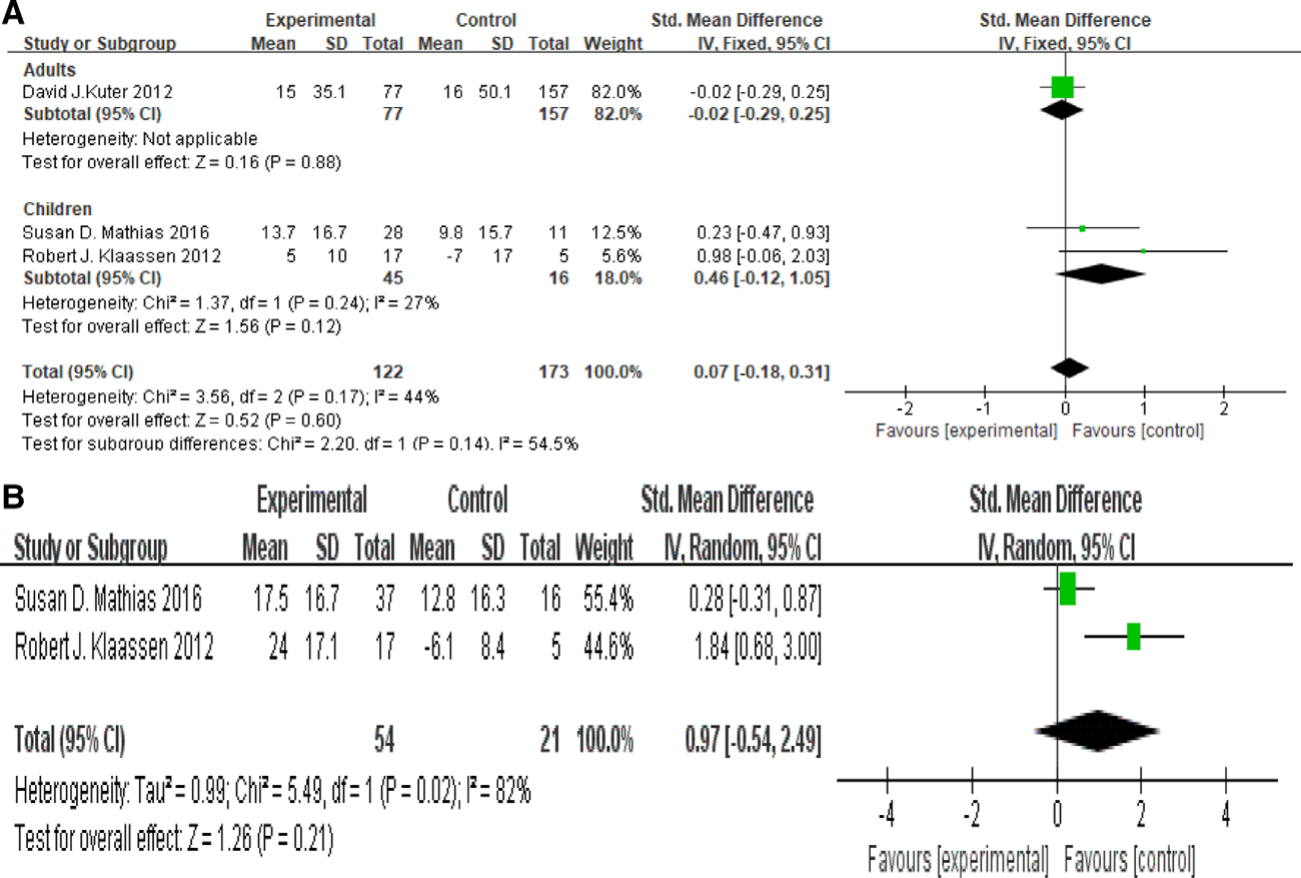
(A): HRQoL in ITP adults and children. (B): ITP children’s parents, HRQoL in each group. HRQoL = health-related quality of life, ITP = immune thrombocytopenia.

Of the 9 articles, 1 reported the change of adult quality of life, and 2 reported the change of child quality of life. In this paper, the subgroups of ITP adults and children were analyzed, and showed the heterogeneity of 2 articles in ITP children was small (*P* = .24, *I*^2^ = 27%), using fixed effect model. However, there was no statistical difference in the changes of child quality of life. As shown in Figure 6A.

There were also 2 articles reported that the change of children’s parents’ quality of life. There was significant heterogeneity among the articles (*P* = .02, *I*^2^ = 91%), so we used the random effect model. Meta-analysis showed that SMD = 0.97, 95% CI [-0.54,2.49], *P*>0.05, Therefore, there was no significant difference in the changes of parents’ quality of life in each group. As shown in Figure 6B.

## 4. Discussion

ITP is a clinically common hemorrhagic disease, and the severity of bleeding in this patient is negatively related to the level of platelet count. In general, the incidence of chronic ITP in adults is 5 to 10/1,00,000, especially women of childbearing age and elderly over 60 years old are the groups with high incidence of this disease. However, most children with acute ITP have a history of infection before onset, and the process can be alleviated by itself.^[15]^ The pathology of ITP is the anti-platelet autoantibodies destroy the platelets and are phagocytized by the monocyte macrophage system in peripheral blood. At the same time, anti-platelet autoantibodies can also enter the bone marrow to cause the megakaryocyte dysfunction or damage. Recent studies have found ITP is a common autoimmune disease and the cellular immune mechanism plays a more important role in its pathogenesis.^[16]^

At present, glucocorticoids and intravenous immunoglobulin have become the first choice for ITP treatment. In addition, immunosuppressants such as cyclosporine and cyclophosphamide, CD20 monoclonal antibodies and thrombopoietin receptor agonists have become new drugs in clinical treatment of ITP in recent years.^[17]^ TPO is an important growth factor that regulates the proliferation and differentiation of megakaryocytes and mediates the production of platelets by megakaryocytes. As the second generation of TPO receptor agonists, Eltrombopag and Romiplostim are more reliable in treatment effect and safety compared with the first generation.^[18]^ Eltrombopag is a non-peptide TPO analogue, which is mainly used for oral treatment of adult chronic ITP patients. Romiplostim is a drug of subcutaneous injection. It can simulate the domain of TPO and compete with endogenous TPO for binding sites on target cells, so as to promote the differentiation of hematopoietic stem cells into megakaryocytes and the production and release of mature platelets in megakaryocytes.^[19]^

Among the 9 articles included, 4 were ITP adults, 5 were ITP children. After that, we analyzed the clinical efficacy, drug safety and HRQoL of Romiplostim in ITP adults and children. The clinical efficacy in Romiplostim mainly includes total platelet response rate and long-term platelet response rate. Platelet response rate refers to the proportion of patients whose platelet count rises to a certain level over a period of time. Some studies have found short-term use of Romiplostim can increase platelet response rate in chronic ITP patients.^[20]^ Our meta-analysis showed that the total platelet response rate and the long-term platelet response rate in each group were relatively small in heterogeneity, indicating that Romiplostim was no significant difference in ITP adults and children. The final results showed that both the total platelet response rate and the long-term platelet response rate of patients in each group were statistically significant, which further indicated that Romiplostim had a certain role and effect in the treatment of chronic ITP adult and children.

In terms of drug safety of Romiplostim in ITP patients, this study mainly analyzed side effects, serious side effects, use of emergency drugs, bleeding events and serious bleeding events in each group. In recent years, studies have found both Eltrombopag and Romiplostim have better drug safety in the treatment of ITP patients.^[21]^ In this study, we found there was no heterogeneity in side effects and use of emergency drugs in each group, and the heterogeneity of serious side effects in each group was small. The results of the final meta-analysis showed the probability of use of emergency drugs in each group was statistically significant, while the other drug safety were not significant, which indicated that the drug safety of Romiplostim were basically similar to those of using placebo in ITP adults and children. Studies have found the adverse reactions caused by Romiplostim in the treatment of ITP patients mainly include headache, fatigue, nasopharyngitis, back pain and limb pain.^[22]^ Further reading and comparison of several articles included in this study found the drug safety of Romiplostim were basically similar to those of using Placebo.

Bleeding events and serious bleeding events is also one of the adverse drug reactions of Romiplostim. Our results showed there was no heterogeneity in severe bleeding events, while the heterogeneity in bleeding events was relatively large. After analyzing the bleeding events in subgroup, it was found the articles heterogeneity among subgroups was large, speculated the probability of bleeding event might be different between ITP adults and children. However, the results of meta-analysis showed these events were not statistically significant in each group, so Romiplostim had the drug safety in treatment of ITP adults and children.

In recent years, researchers have further increased the study of HRQoL in ITP patients based on the efficacy of Romiplostim, paying more attention to patients’ quality of life and spiritual feelings during treatment. The HRQoL for ITP adults and children was completed by the ITP-patient assessment questionnaire during the treatment period, mainly included symptoms, disturbance, activities, mental health, fear, overall quality of life and social quality of life. However the HRQoL for ITP children is the Kids’ ITP Tools, which is mainly divided into 2 parts: children’s self-report and parents’ influence.^[23]^ In this study, a total of 3 articles related to HRQoL were included, 1 article for ITP adults and 2 articles for children. Because ITP adults and children may have differences in the questionnaire pattern of HRQoL, we analyzed the changes in HRQoL of ITP adults and children respectively. It showed that although there was a certain trend in Romiplostim on ITP children’s HRQoL with these 2 articles, but the results were not statistically significant. Later, this study further analyzed the HRQoL of ITP children’s parents in these 2 articles. It showed that although there was a certain trend in Romiplostim on ITP children’s parents HRQoL with these 2 articles, but the results were not statistically, significant. So we can speculated that Romiplostim has a certain impact on HRQoL of ITP children and their parents. However, the number of articles we included is relatively small. It is expected that more high-level RCTs in Romiplostim on HRQoL of ITP adults and children will appear in the future.

Therefore, this study preliminarily concluded that Romiplostim can increase the platelet response rate in ITP adults and children, which has certain clinical efficacy and high drug safety. This meta-analysis still has some shortcomings such as a small number of articles, which need to be included in larger sample size and higher quality RCTs, especially the study on HRQoL of ITP adults and children and ITP children’s parents by Romiplostim. Looking forward to more high-quality RCTs in the future to provide a better basis for studies on clinical efficacy, drug safety and HRQoL of Romiplostim in ITP adults and children.

## Acknowledgments

This work was funded by the National Natural Science Foundation of China (81770118, 81970116).

## Author contributions

**Conceptualization:** Zonghong Shao.

**Data curation:** Ningyuan Ran.

**Funding acquisition:** Zonghong Shao.

**Project administration:** Ting Wang.

**Writing – original draft:** Xin He.

**Writing – review & editing:** Xin He.
